# Identification of LZAP as a New Candidate Tumor Suppressor in Hepatocellular Carcinoma

**DOI:** 10.1371/journal.pone.0026608

**Published:** 2011-10-19

**Authors:** Jing-jing Zhao, Ke Pan, Jian-jun Li, Yi-bing Chen, Ju-gao Chen, Lin Lv, Dan-dan Wang, Qiu-zhong Pan, Min-shan Chen, Jian-chuan Xia

**Affiliations:** 1 State Key Laboratory of Oncology in Southern China and Department of Experimental Research, Sun Yat-sen University Cancer Center, Guangzhou, People's Republic of China; 2 Department of Hepatobiliary Oncology, Sun Yat-sen University Cancer Center, Guangzhou, People's Republic of China; University of Illinois at Chicago, United States of America

## Abstract

**Background:**

LZAP was isolated as a binding protein of the Cdk5 activator p35. LZAP has been highly conserved during evolution and has been shown to function as a tumor suppressor in various cancers. This study aimed to investigate LZAP expression and its prognostic value in hepatocellular carcinoma (HCC). Meanwhile, the function of LZAP in hepatocarcinogenesis was further investigated in cell culture models and mouse models.

**Methods:**

Real-time quantitative PCR, western blot and immunohistochemistry were used to explore LZAP expression in HCC cell lines and primary HCC clinical specimens. The functions of LZAP in the proliferation, colony formation, cell cycle, migration, invasion and apoptosis of HCC cell lines were also analyzed by infecting cells with an adenovirus containing full-length LZAP. The effect of LZAP on tumorigenicity in nude mice was also investigated.

**Results:**

LZAP expression was significantly decreased in the tumor tissues and HCC cell lines. Clinicopathological analysis showed that LZAP expression was significantly correlated with tumor size, histopathological classification and serum α-fetoprotein (AFP). The Kaplan–Meier survival curves revealed that decreasing LZAP expression was associated with poor prognosis in HCC patients. LZAP expression was an independent prognostic marker of overall HCC patient survival in a multivariate analysis. The re-introduction of LZAP expression in the HepG2 and sk-Hep1 HCC cell lines significantly inhibited proliferation and colony formation in the HCC cells and induced G1 phase arrest and apoptosis of the HCC cells in vitro. Restoring LZAP expression in the HCC cell lines also inhibited migration and invasion. In addition, experiments with a mouse model revealed that LZAP overexpression could suppress HCC tumorigenicity in vivo.

**Conclusions:**

Our data suggest that LZAP may play an important role in HCC progression and could be a potential molecular therapy target for HCC.

## Introduction

Hepatocellular carcinoma (HCC) is currently the fifth most common solid tumor worldwide and the fourth leading cause of cancer-related death in many countries, especially in East Asia [1]–[3]. Even with aggressive treatment, HCC usually has a poor prognosis, with a 5-year survival rate as low as 25%–39% after common treatments, such as surgery, chemotherapy, and radiotherapy [4]. The poor prognosis of HCC is also caused by its poorly differentiated phenotype, portal venous invasion, and intrahepatic metastasis [5]. To improve patient outcomes, it is clinically important to find efficient new targets for the early diagnosis and effective treatment of HCC [6]. Hepatocarcinogenesis is a multifactorial and multistep process that involves activating oncogenes and inactivating tumor suppressor genes in different stages of HCC progression [7]–[9]. Clarifying and investigating the roles of the genes involved in HCC development will contribute to our understanding of the mechanisms of hepatocarcinogenesis [6].

LZAP, also known as the Cdk5rap3 or C53 protein, has been highly conserved throughout evolution and was originally isolated as a binding protein of the Cdk5 activator p35. Human LZAP consists of 506 amino-acid residues without well-defined domains, except for a small leucine zipper region [10]. Recently, LZAP has been identified as an ARF-binding protein and was found to have numerous tumor-suppressor functions [11]. LZAP overexpression potentiates DNA damage-induced cell death by etoposide and x-ray irradiation and renders cells susceptible to multiple genotoxins by modulating the G2/M checkpoint [12]. In addition, it has been shown that LZAP acts as a novel tumor suppressor in primary head and neck cancers by specifically inhibiting NF-κB signaling and that decreased LZAP expression promotes cellular transformation, xenograft tumor growth, and xenograft tumor vascularity [8]. It has also been shown that the tumor suppressor protein C53 antagonizes checkpoint kinases to promote cyclin-dependent kinase 1 activation [10]. Recently, LZAP has been shown to inhibit cell invasion through binding to NLBP, another tumor suppressor [13]. Until now, however, LZAP expression and its prognostic value in HCC patients have not been examined, and the functional role of LZAP in the pathogenesis and tumorigenicity of HCC have not been determined.

In the present study, we investigated the expression of LZAP in primary HCC using real-time quantitative RT-PCR, western blotting and immunohistochemistry. We also identified the relationship between LZAP expression and several clinicopathological features of HCC and evaluated the prognostic value of LZAP expression for the survival of HCC patients. Furthermore, we explored the role of LZAP in HCC tumor progression using cell proliferation, colony formation, cell cycle, migration, invasion, and apoptosis assays in vitro. Finally, we examined the role of LZAP on HCC tumorigenicity in injectable mouse models.

## Results

### LZAP mRNA and protein expression in primary HCC tissue samples and HCC cell lines

**Figure 1:**
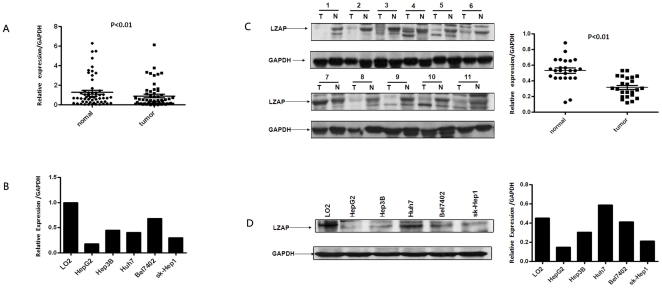
The expression of LZAP mRNA and protein in the human primary HCC surgical specimens and HCC cell lines, as evaluated by real-time quantitative PCR and western blotting. (**A**) The relative mRNA expression of LZAP was lower in 57 HCC tumor tissues than in matched adjacent non-tumorous tissues (p<0.01). (**B**) The LZAP mRNA expression in human hepatocellular carcinoma cell lines was down-regulated in the HepG2, Hep3B, Huh7, Bel7402 and sk-Hep1 cells, particularly in the HepG2 and sk-Hep1cells, compared with the normal liver cell line LO2. (**C**) The LZAP protein expression was lower in the tumor tissues than in matched adjacent non-tumorous tissues (p<0.01). (**D**) The LZAP protein levels were significantly lower in the HepG2 and sk-Hep1 cells than in the normal liver cell line LO2.

A real-time quantitative PCR was performed on 57 paired clinical samples from HCC patients (tumor tissues and matched adjacent non-tumor liver tissues) and hepatocellular carcinoma cell lines to determine their LZAP mRNA levels. In the clinical samples, the LZAP expression was lower in the tumor tissues than in the matched adjacent non-tumor liver tissues (p<0.01, Fig. 1A). Furthermore, the HepG2, Hep3B, Huh7, Bel7402 and sk-Hep1 HCC cell lines showed decreased LZAP transcript levels relative to the LO2 normal liver cell line. Additionally, LZAP expression was significantly lower in the HepG2 and sk-Hep1 cells (Fig. 1B).

To investigate whether LZAP was also reduced at the protein level, western blotting was performed on 24 HCC clinical samples, the corresponding adjacent non-tumorous liver tissues and the HCC cell lines. As shown in Figure 1C, LZAP protein expression was lower in the tumors (p<0.01), consistent with the results of real-time quantitative PCR. Likewise, LZAP protein expression was decreased in the HepG2 and sk-Hep1 cells compared to the LO2 cells (Fig. 1D).

### Immunohistochemical analysis of LZAP expression in HCC clinical samples and its relationship to clinicopathological parameters

**Figure 2:**
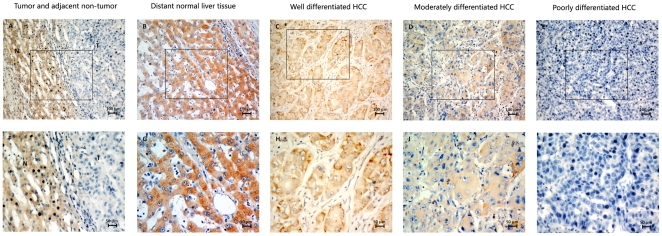
Immunohistochemical analysis of the LZAP protein expression in the primary hepatocellular carcinoma surgical specimens. (**A**) **and** (**F**) Immunostaining of an HCC tumor and the adjacent non-tumorous area. (**B**) **and** (**G**) Normal liver tissue distant from the tumor, scored as LZAP (+++). (**C**) **and** (**H**) Well-differentiated HCC, scored as LZAP (++). (**D**) **and** (**I**) Moderately differentiated HCC, scored as LZAP (+). (**E**) **and** (**J**) poorly differentiated HCC, scored as LZAP (−). N: non-tumor tissue; T: tumor tissue (A–E with 200× magnification; F–J with 400× magnification).

LZAP expression was investigated in 126 HCC surgical specimens using immunohistochemical staining; 76 (60.3%) cases showed low LZAP expression (LZAP− or LZAP+), and 50 (39.7%) cases exhibited high LZAP expression (LZAP++ or LZAP+++) (Table 1). In the positive cases, LZAP was detected in the cytoplasm of the cells (Fig. 2G, H and I). LZAP expression was also observed in normal liver tissues distant from the tumors (Fig. 2B and G). In cases with adjacent hyperplastic tissue, we often observed a sharp contrast between the infiltrative areas of negative staining representing the tumor and the adjacent, positively stained non-tumor tissue (Fig. 2A and F). The relationship between LZAP expression and various clinicopathological parameters is described in Table 1. LZAP expression was significantly correlated with tumor size (p = 0.040), histological differentiation (p = 0.001), and serum AFP (P = 0.006). Well-differentiated cases showed strongly positive LZAP expression (Fig. 2C and H), moderately-differentiated cases showed weakly positive expression (Fig. 2D and I), and the most poorly differentiated cases often showed no detectable LZAP expression (Fig. 2E and J). There was no statistically significant difference in LZAP expression by age, gender, liver cirrhosis, HBV, recurrence, or distant metastasis.

**Table 1:** Relationship between LZAP expression and clinicopathological features of 126 patients with hepatocellular carcinoma.

Clinicopathologic variables	number of each group	LZAP expression		*p* value
		low	high	
All cases	126	76	50	
Age(years)				0.216
<50	64	42	22	
≥50	62	34	28	
Gender				0.379
Male	115	68	47	
Female	11	8	3	
Tumor size(cm)				0.040^a^
<5	54	27	27	
≥5	72	49	23	
Histilogical differentiation				0.001^a^
Well	21	7	14	
Moderate	61	34	27	
Poor	44	35	9	
Liver cirrhosis				0.185
No	57	38	19	
Yes	69	38	31	
HBV				0.722
Negative	16	9	7	
Positive	110	67	43	
Serum AFP				0.006^a^
<25 ug/l	38	16	22	
≥25 ug/l	88	60	28	
Recurrence				0.900
No	94	57	37	
Yes	32	19	13	
Distant Metastasis				0.656
No	108	66	42	
Yes	18	10	8	

a *p* value<0.05.

### LZAP expression and patient survival

**Figure 3:**
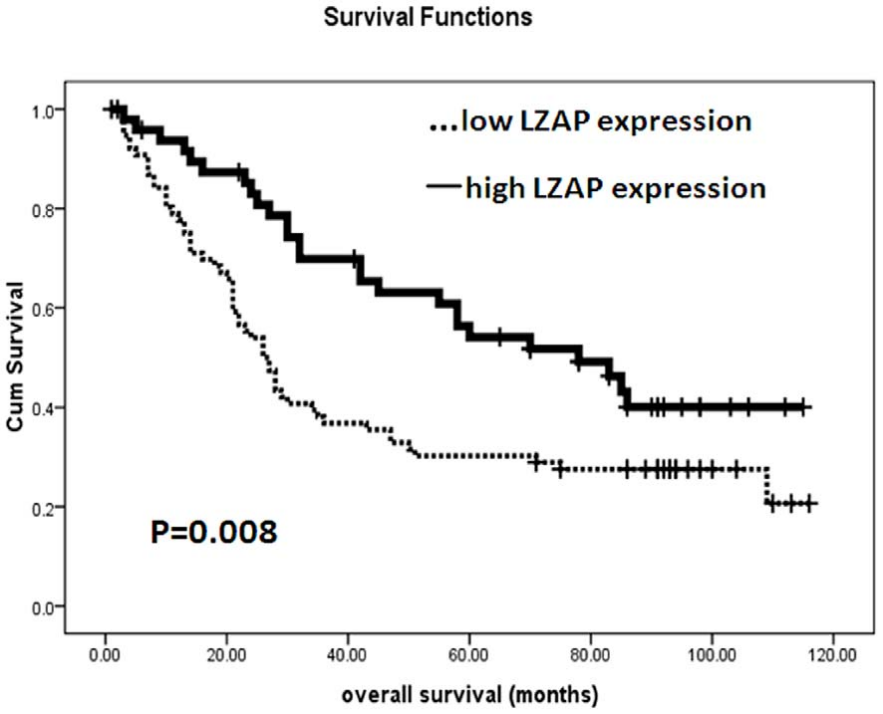
The Kaplan–Meier survival analysis of the primary HCC patients (n = 126) with high LZAP expression (n = 50) and low LZAP expression (n = 76) after surgical resection. Based on their LZAP immunostaining scores, the HCC patients were divided into low-LZAP expression (LZAP− or LZAP+) and high-LZAP expression (LZAP++ or LZAP+++) groups. The survival rate of the patients in the low-LZAP group was significantly lower than that of the patients in the high-LZAP group (p = 0.008 for the log-rank test).

The prognostic value of LZAP for overall survival in HCC patients was evaluated by comparing the patients with high and low LZAP expression. According to a Kaplan–Meier survival analysis, low LZAP expression was significantly associated with poor prognosis. The HCC patients with low LZAP expression had obviously lower overall survival rates than did those with high LZAP expression (Fig. 3, p = 0.008 for the log rank test).

### Univariate and multivariate analyses of prognostic variables in HCC patients

Further univariate and multivariate analyses were conducted using a Cox proportional-hazards model to examine the impact of LZAP expression and other clinical pathological parameters in HCC patients. LZAP expression, histological grade, serum AFP, and recurrence were significant prognostic factors in the univariate analysis (Table 2). Multivariate Cox regression analyses showed that LZAP was an independent predictor. Thus, LZAP expression may be useful for predicting the overall survival of HCC patients (p = 0.043, Table 2).

**Table 2:** Univariate and multivariate analysis of overall survival in hepatocellular carcinoma.

Variables	Univariate analysis			Multivariate analysis		
	HR	95% CI	*p* value	HR	95% CI	*p* value
LZAP	0.538	0.337–0.858	0.009^a^	0.608	0.375–0.984	0.043^a^
Age	0.886	0.574–1.366	0.583			
Gender	0.702	0.305–1.613	0.404			
Tumor size	1.413	0.906–2.204	0.127			
Histologic grade	1.772	1.272–2.470	0.001^a^	1.425	0.994–2.044	0.054
Liver Cirrhosis	0.700	0.454–1.081	0.108			
HBsAg status	2.030	0.933–4.414	0.074			
Serum AFP	2.271	1.329–3.881	0.003^a^	1.611	0.898–2.890	0.110
Recurrence	1.616	1.008–2.591	0.046^a^	1.626	1.004–2.632	0.048^a^
Distant Metastasis	1.504	0.858–2.638	0.154			

*HR* Hazard ratio, *CI* confidence interval.

a *p* value<0.05.

### LZAP inhibits the viability of HepG2 and sk-Hep1 cell lines

**Figure 4:**
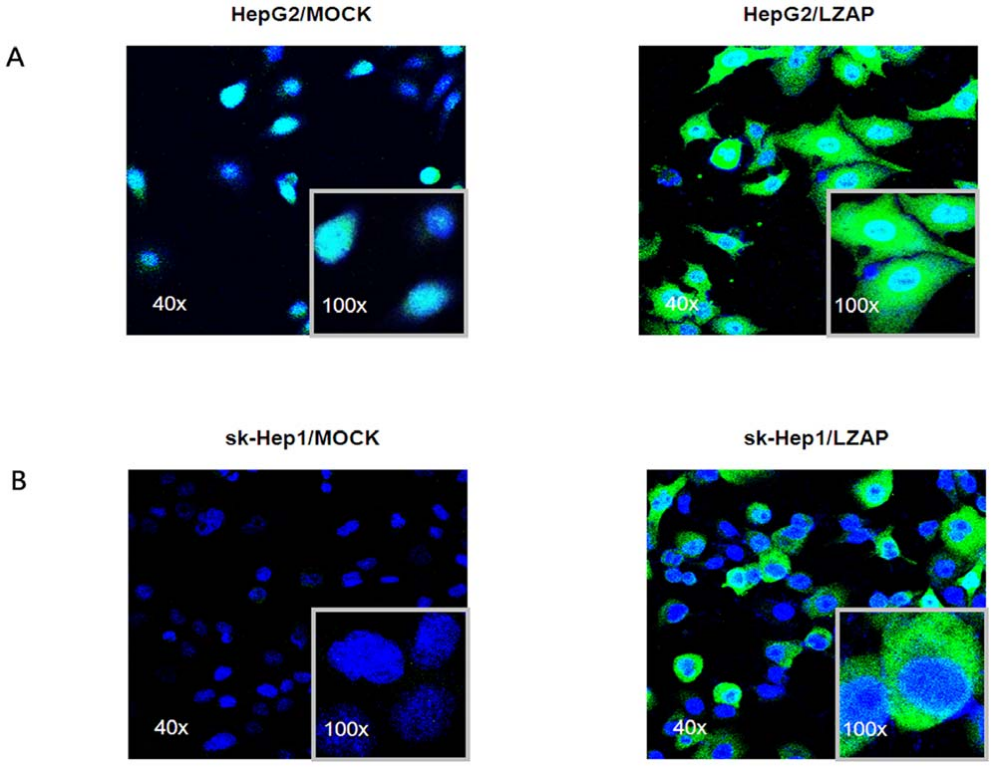
LZAP protein expression in the HepG2 and sk-Hep1 cells infected with Ad-control and Ad-LZAP. (**A**) The LZAP expression (green, mainly in the cytoplasm) in the HepG2 cells infected with Ad-LZAP was strikingly higher than that that in the cells infected with the Ad-control. (**B**) The LZAP expression (green, in the cytoplasm) was also higher in the sk-Hep1 cells infected with Ad-LZAP than in the sk-Hep1 cells infected with the Ad-control. The cell nuclei (blue) were stained with DAPI. 40× magnification. Inset: 100× magnification.

**Figure 5:**
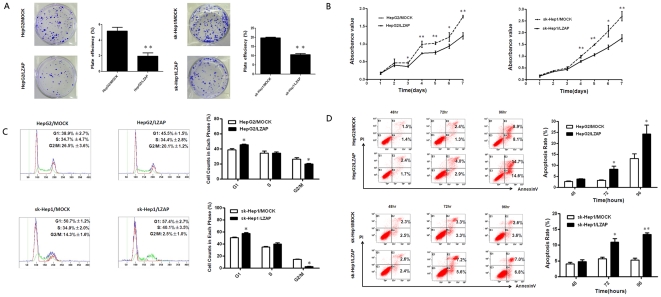
Overexpression of LZAP decreases HCC cell viability. (**A**) LZAP inhibited colony formation in the HepG2 and sk-Hep1 cells. The images are shown on the left and on the right, and the mean ± SD of the foci for each group are shown. The experiments were performed in triplicate. The p values were calculated using the Student's t-test. (**B**) The MTS assay, showing that LZAP suppressed the proliferation of the HepG2 and sk-Hep1 cells. (**C**) The effect of LZAP overexpression on the cell cycle. LZAP overexpression caused G1 arrest in the HepG2 and sk-Hep1 cells. (**D**) The effect of LZAP overexpression on apoptosis in the HepG2 and sk-Hep1 cells at 48 hours, 72 hours, and 96 hours after the adenovirus infection. LZAP induced cell apoptosis, especially at 72 hours and 96 hours. *p<0.05 versus Ad-control; **<0.01 versus Ad-control.

To evaluate the effect of LZAP on cell viability, the HepG2 and sk-Hep1 cells with low LZAP expression were infected with Ad-LZAP and Ad-control. LZAP expression in the infected cells was confirmed by immunofluorescence (Fig. 4) and western blotting (Fig. S1). A colony formation assay was used to explore the ability of LZAP to inhibit tumor cell growth. Compared with the Ad-control-infected cells, the HepG2 and sk-Hep1 cells infected with Ad-LZAP showed a significant reduction in colony formation ability (Fig. 5A). Cell proliferation assays revealed that the growth rate of the HepG2 and sk-Hep1 cells infected with Ad-LZAP was significantly lower than that of the HepG2 and sk-Hep1 cells infected with the Ad-control (Fig. 5B). We then examined the possible effects of LZAP expression on the cell cycle by flow cytometric analysis. We found that overexpressing LZAP in the HCC cell lines induced G1 phase arrest (Fig. 5C). Furthermore, we investigated the role of LZAP in the apoptosis of HCC cells. More apoptotic cells were found in the LZAP-overexpressing HCC lines than in control lines (Fig. 5D).

To further elucidate the biological functions of LZAP in HCC, we used siRNA to knockout LZAP expression in the HepG2 and sk-Hep1 cells infected with Ad-LZAP. Western blotting showed that siLZAP-3# had the highest knockout efficiency of the three siRNAs tested (Fig. S2). Therefore, siLZAP-3# was used for all the subsequent experiments. Silencing LZAP expression in the HepG2 and sk-Hep1 cells infected with Ad-LZAP increased cell proliferation and colony formation to levels similar to those of the HepG2 and sk-Hep1 infected with the Ad-control (Fig. S3).

### LZAP inhibits cell migration and invasion in the HepG2 and sk-Hep1 cell lines

**Figure 6:**
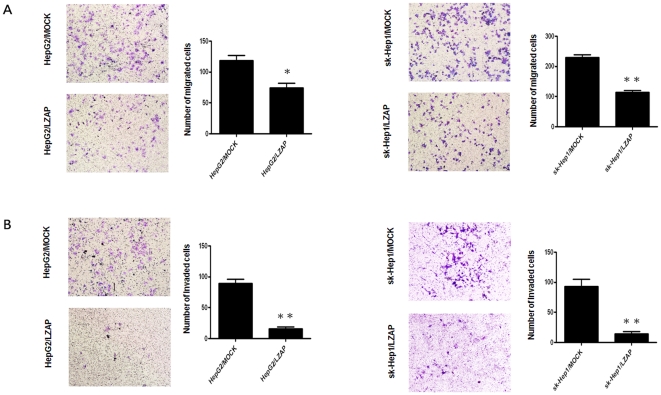
Transwell migration assays and Matrigel invasion assays of HepG2 and sk-Hep1 cells infected with Ad-LZAP and Ad-control. Images are shown on the left (magnification: 100×), and the quantification of 10 randomly selected fields is shown on the right. The values shown are expressed as the mean ± SD of three independent experiments. The p values were calculated using Student's t-test. (**A**) LZAP inhibited cell migration in the HepG2 and sk-Hep1 cells. (**B**) LZAP also significantly inhibited cell invasion by the HepG2 and sk-Hep1 cells. *p<0.05 versus Ad-control; **<0.01 versus Ad-control.

We employed a transwell assay to evaluate the effects of LZAP expression on cell migration and invasion. HepG2 and sk-Hep1 cells infected with Ad-LZAP migrated into the lower compartment of the migration chamber significantly less frequently than did cells infected with the Ad-control (Fig. 6A). Consistent with the migration assay results, LZAP also significantly inhibited cell invasion through a Matrigel-coated membrane (Fig. 6B).

### LZAP suppresses tumorigenicity of HCC in vivo

**Figure 7:**
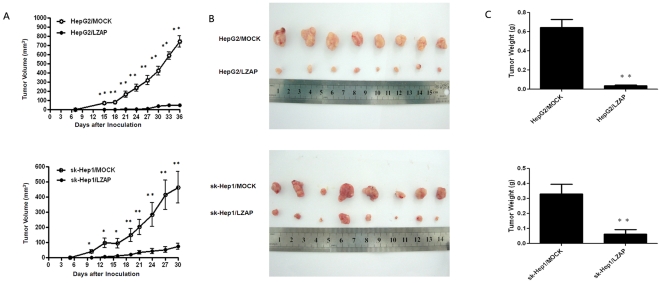
LZAP suppresses the tumorigenicity of HCC in vivo. HepG2 or sk-Hep1 cells infected with Ad-control and Ad-LZAP were injected into nude mice, as described in the Materials and Methods section. The tumor volumes were measured every 3 days. At the end of the experiment, the animals were sacrificed and the tumors were excised for volume and weight measurement. (**A**) The tumor growth curves for each group. The tumor growth rate was reduced in the tumors that overexpressed LZAP. (**B**) Photographs of dissected tumors from the nude mice. The final tumor volumes were smaller in the HepG2-LZAP and sk-Hep1-LZAP groups than in the control group. (**C**) The tumor weights of each group. The final tumor weights were reduced in the tumors that overexpressed LZAP. The data are presented as mean ± SD. *p<0.05 versus Ad-control; **<0.01 versus Ad-control.

To assess the role of LZAP in tumor growth in vivo, the HepG2 or sk-Hep1 cells infected with Ad-control and Ad-LZAP were injected subcutaneously into nude mice. The results showed that LZAP overexpression in the HCC cells significantly delayed tumor growth in the mice (Fig. 7A). Furthermore, the mean tumor volume in the LZAP overexpressed group at the end of observation was significantly smaller than that of the control group (49.37 mm^3^ vs. 743.57 mm^3^ for HepG2, 74.48 mm^3^ vs. 464.81 mm^3^ for sk-Hep1; Fig. 7A and B). Accordingly, the mean tumor weight in the LZAP overexpressed group was markedly lower than in the control group (0.037 g vs. 0.646 g for HepG2, 0.062 g vs. 0.329 g for sk-Hep1; Fig. 7C).

## Discussion

In the present study, we used a relatively large series of clinical tissue samples to explore the role of LZAP in HCC for the first time. We examined LZAP mRNA and protein expression in paired primary HCC samples and HCC cell lines using real-time quantitative PCR and western blotting. We found that LZAP expression was down-regulated at both the transcriptional and translational levels in most primary HCC tumor tissues and HCC cell lines. Consistent with these observations, immunohistochemical analyses also showed that LZAP expression was decreased in most HCC tumor tissues compared with the corresponding non-tumorous liver tissues. These results indicated that down-regulated LZAP expression may play a role in HCC development.

In the immunohistochemical analysis, decreased LZAP expression in HCC was significantly associated with tumor size, histological differentiation, and serum AFP. The relationship between low LZAP expression and larger tumor size suggested that the decline in LZAP expression may help facilitate the rapid expansion of the tumor. Additionally, most of the well-differentiated HCC samples were positive for LZAP expression, but LZAP expression was profoundly weaker in the moderately and poorly differentiated tumor samples. Thus, decreased LZAP expression is correlated with poor differentiation in HCC cells and may further promote HCC progression.

Our Kaplan-Meier survival analysis revealed that low LZAP expression was significantly linked to poor prognosis after surgical resection in the HCC patients (p = 0.008). Furthermore, LZAP expression was an independent prognostic factor for overall survival in the multivariate analysis. These results suggest that LZAP can serve as a new predictor of prognosis in HCC patients after surgical resection.

Based on the low LZAP expression in the HepG2 and sk-Hep1 HCC cell lines, we transfected full-length LZAP into these cells to further examine the mechanism by which it suppresses HCC progression. Restoring LZAP expression significantly suppressed cell proliferation and colony formation. In parallel experiments, we also found that LZAP overexpression suppressed tumor growth in injectable mouse models. Consistent with our results, a previous study has shown that decreased LZAP expression in human head and neck squamous cell carcinomas promotes cellular transformation and xenograft tumor growth [8]. LZAP overexpression also led to G1 phase cell cycle arrest and induced apoptosis of HCC cells, indicating that LZAP suppresses HCC tumorigenicity by inducing cell cycle arrest and apoptosis. Wang et al. have also confirmed that LZAP expression, in addition to ARF, increases the percentage of cells in the G1 phase of the cell cycle [11]. In our study, we also found that LZAP overexpression inhibited the migration and invasion of the HepG2 and sk-Hep1 cells, suggesting that LZAP may suppress tumor metastasis. Consistent with our results, Wang et al. have found that losing LZAP increases cellular invasion [8]. This finding strengthens the hypothesis that LZAP acts as an HCC tumor suppressor.

In conclusion, our study found that LZAP expression was down-regulated in the majority of the HCC clinical tissue specimens at both mRNA and protein levels and that low LZAP expression may be correlated with unfavorable prognosis in HCC patients. Cell culture studies confirmed that LZAP overexpression inhibited cell proliferation, colony formation, migration/invasion and induced cell cycle arrest, apoptosis in HCC cell lines. The mouse model experiments revealed that LZAP overexpression significantly inhibited the tumor growth. Our findings provide evidence that LZAP could be a novel prognostic biomarker and a new molecular therapy target for HCC.

## Materials and Methods

### Cell lines and culture conditions

The HepG2, Hep3B and sk-Hep1 HCC cell lines were obtained from the American Type Culture Collection (ATCC). The LO2, Bel7402 and HEK293 cell lines were obtained from the Committee of Type Culture Collection of the Chinese Academy of Sciences (Shanghai, China). The Huh7 cell line was obtained from the RIKEN cell bank (Ibaraki, Japan). The LO2, Bel7402 and sk-Hep1 cells were cultured in RPMI 1640 supplemented with 10% heat-inactivated FBS (fetal bovine serum) and 1% penicillin-streptomycin. The HepG2, Hep3B, Huh7 and HEK293 cells were cultured in DMEM (Dulbecco modified Eagle medium) supplemented with 10% heat-inactivated FBS (fetal bovine serum) and 1% penicillin-streptomycin. All cells were incubated at 37°C in a humidified chamber containing 5% CO2.

### Patients and tumor tissue samples

A total of 126 human primary HCC tissues and matched control tissues were obtained from patients who underwent hepatectomy at the Sun Yat-sen University Cancer Center between 2001 and 2004. None of these patients had received preoperative chemotherapy or radiotherapy. The follow-up data from the HCC patients in this study were available and complete. The postoperative follow-up occurred at our outpatient department and included clinical and laboratory examinations every 3 months for the first 2 years, every 6 months during the third to fifth years, and annually for an additional 5 years or until patient death, whichever occurred first. Overall survival, which was defined as the time from the operation to patient death or the last follow-up, was used as a measure of prognosis. Both the tumor and the corresponding non-tumor tissues not less than 2 cm away from the HCC were sampled, and the diagnosis was confirmed by pathological examination. After surgical resection, the matched fresh tissues were immediately immersed in RNAlater (Ambion, Inc., USA), kept at 4°C overnight, then stored at −80°C until the RNA isolation. All the tissue samples were fixed in 10% formalin and embedded in paraffin, and consecutive 2 µm sections were cut. Histological types were assigned according to the WHO classification criteria. This study was approved by the Ethics Committee of the Sun Yat-sen University Cancer Center, and written informed consent was obtained from each patient.

### RNA extraction and real-time quantitative PCR

Total RNA was extracted using TRIzol solution (Invitrogen, USA) according to the manufacturer's instruction. RNase-free DNase I was used to remove DNA contamination. The total RNA concentration and quantity were assessed by absorbency at 260 nm using a Nanodrop spectrophotometer (ND-1000, Thermo Scientific, USA). The first-strand cDNA synthesis was performed using 2 µg of total RNA and M-MLV reverse transcriptase according to the manufacturer's instructions (Promega, USA). The resulting cDNAs were subjected to real-time PCR analysis to evaluate the relative expression levels of LZAP and GAPDH (an internal control) using the following primers: 5′- TCTGGGTCCTACATTCACTACTTTC-3′ (F) and 5′-CTCCTGCCAATCCTTCATCC-3′ for LZAP; and 5′-CTCCTCCTGTTCGACAGTCAGC-3′ (F) and 5′- CCCAATACGACCAAATCCGTT-3′ for GAPDH. Each 15 µl of reaction volume contained 0.5 µl of cDNA that was synthesized as above, 7.5 µl of 2× SYBR Green master mix (Invitrogen, USA), and 200 nM of each pair of oligonucleotide primers described above. The cycling parameters began with 95°C for 10 minutes, followed by 45 cycles of 90°C for 30 seconds and 60°C for 60 seconds, followed by a melting curve analysis. Ct was measured during the exponential amplification phase, and the amplification plots were analyzed using the software provided with the instrument (SDS 2.0). The relative expression levels of the target gene were normalized to that of the internal control gene, GAPDH. The data were analyzed using the comparative threshold cycle (2^−ΔΔCT^) method.

### Protein extraction and western blotting analysis

The frozen HCC samples, including the tumor tissue, non-tumor control tissue and cells from the HCC cell lines (LO2, HepG2, Hep3B, Huh7, Bel7402 and sk-Hep1), were homogenized in a RIPA lysis buffer, and the lysates were cleared by centrifugation (14,000 rpm) at 4°C for 30 minutes. Approximately 40 µg of protein sample were run on a 12% SDS-PAGE gel and transferred to PVDF membranes. After blocking the non-specific binding sites for 60 minutes with 5% non-fat milk, the membranes were incubated with primary monoclonal antibodies against LZAP (at a 1∶1000 dilution) or GAPDH (at a 1∶10000 dilution) overnight at 4°C. Next, the membranes were subjected to three 15 minute washes with TBST and then incubated with HRP-conjugated secondary antibody (at a 1∶2000 dilution) for 45 minutes at room temperature. The membrane was washed three more times with TBST and developed using an enhanced chemiluminescence system (ECL, Cell Signaling Technologies).

### Immunohistochemistry and immunofluorescence

Paraffin-embedded tissue blocks were sectioned for immunohistochemistry. The sections were deparaffinized and rehydrated with graded ethanol. For the antigen retrieval, the slides were immersed in EDTA (1 mmol/L, pH 8.0) and boiled for 15 minutes in a microwave oven. After rinsing with PBS, the endogenous peroxidase was blocked with 0.3% hydrogen peroxide for 15 minutes at room temperature. The slides were incubated with the primary antibody (mouse anti-LZAP monoclonal antibody, Santa Cruz Biotechnology, USA, at a 1∶500 dilution) overnight in a humidified chamber at 4°C. The sections were washed three times with PBS, incubated with horseradish peroxidase-conjugated secondary antibody (Envision™ Detection Kit, GK500705, Gene Tech) at 37°C for 30 minutes, and then washed three more times with PBS. Finally, 3, 3′-diaminobenzidine tetrahydrochloride (DAB) was used for signal development, and the sections were counterstained with 20% hematoxylin. The slides were dehydrated, cleared and evaluated. Each sample was incubated with an isotypic antibody dilution under the same experimental conditions as the negative control.

The HepG2 or sk-Hep1 cells infected with the Ad-control or Ad-LZAP were plated on glass coverslips. At 48 hours post-infection, the coverslips were washed extensively in PBS and fixed with 4% paraformaldehyde. After rinsing with PBS, the cells were permeabilized with 0.2% Triton X-100 in PBS for 10 minutes. The coverslips were then washed and blocked with 1% BSA in PBS for 60 minutes. The slides were incubated with mouse anti-LZAP monoclonal antibody (Santa Cruz Biotechnology, USA, at a 1∶100 dilution) in PBS with 0.2% Triton X-100 and 0.1% BSA at room temperature for 1 hour. The slides were then washed extensively with PBS and treated with fluorescein (FITC)-conjugated goat anti-mouse secondary antibody (Santa Cruz Biotechnology, USA) for 30 minutes. After further washing, the slides were labeled with DAPI and images were acquired using a Laser Scanning Confocal Microscope.

### Semi-quantitative method

The total LZAP immunostaining score was calculated as both the percentage of positively stained tumor cells and the staining intensity. The percent positivity was scored as “0” (<5%, negative), “1” (5%–25%, sporadic), “2” (25%–50%, focal), or “3” (>50%, diffuse). The staining intensity was scored as “0” (no staining), “1” (weakly stained), “2” (moderately stained), or “3” (strongly stained). Both the percentage of positive cells and the staining intensity were evaluated under double-blind conditions. The LZAP immunostaining score was calculated as the percentage positive score × the staining intensity score and ranged from 0 to 9. We defined the LZAP expression levels as follows: ‘−’ (score 0–1), ‘+’ (score 2–3), ‘++’ (score 4–6) and‘+++’ (score >6). Based on the LZAP expression levels, the HCC patients were divided into two groups: the low LZAP expression group (LZAP− or LZAP+) and the high LZAP expression group (LZAP++ or LZAP+++).

### RNA oligonucleotides and cell transfections

The siRNAs for the LZAP knockouts were synthesized by GenePharma (Shanghai, China). The siRNA sequences were as follows: siLZAP-1#, sense = 5′- GGAGAUUAUAGCUCUGUAUTT-3′ and antisense = 5′- AUACAGAGCUAUAAUCUCCTT- 3′; siLZAP-2#, sense = 5′- GAGAUCCCCUCACUGAAGATT -3′ and antisense = 5′- UCUUCAGUGAGGGGAUCUCTT -3′; siLZAP-3#, sense = 5′- CCCUGACACUGCUUGAAUATT- 3′ and antisense = 5′- UAUUCAAGCAGUGUCAGGGTT -3′; and negative control (NC), sense = 5′- UUCUCCGAACGUGUCACGUTT-3′and antisense = 5′- ACGUGACACGUUCGGAGAATT -3′.

The HepG2 and sk-Hep1 cells were infected with Ad-LZAP and transfected with 20 µM siLZAP or NC 48 hours later. The transfection was performed using the Lipofectamine RNAi MAX reagent (Invitrogen, USA) according to the manufacturer's protocol.

### Recombinant adenovirus construction and tumor cell infection

The LZAP-recombined adenoviral expression vector and the control vector were constructed by the rapid BP/LR reaction in the Gateway cloning system (Invitrogen, USA) according to the manufacturer's instructions. PacI enzyme-linearized adenoviral vectors were transfected into the HEK293 cells using Lipofectamine 2000 (Invitrogen, USA). At 10–13 days after the transfection, when an approximately 80% cytopathic effect (CPE) was observed in the HEK293 cells, the adenovirus-containing HEK293 cells and media were collected. Three freeze/thaw cycles followed by centrifugation were used to prepare the viral lysates. The Ad-LZAP and Ad-control titers were measured with an Adenovirus Titer Immunoassay Kit (Innogent, China). The recombinant adenoviruses were stored at −80°C for use. The HepG2 and sk-Hep1 cells were cultured in 6-well plates and infected with adenovirus (Ad-LZAP and Ad-control) at a multiplicity of infection (MOI) of 200.

### Colony formation assay

The cells infected with Ad-LZAP and Ad-control (1×10^3^) were plated in each well of a 6-well plate. The surviving colonies (>50 cells) were counted with crystal violet staining after two weeks of culture. Colony-forming efficiency (CFE %) was defined as the ratio of the number of colonies formed in culture to the number of cells inoculated. This experiment was performed in triplicate.

### Proliferation assay

The MTS cell proliferation assay was used to evaluate the growth rate of the cells infected with Ad-LZAP and Ad-control. The cells were seeded in 96-well plate at a density of 5×10^3^ per well. The growth rate was detected using the MTS cell proliferation kit according to the manufacturer's instructions (Promega, USA). Three independent experiments were performed.

### Cell cycle assay

The cell cycle analysis was performed at 48 hours after the cells were infected. The HepG2 or sk-Hep1 cells infected with Ad-LZAP and Ad-control were washed twice with ice-cold PBS and fixed with ice-cold 75% ethyl alcohol at −20°C for one hour. After two PBS washes, the cells were resuspended in 400 µl of ice-cold PBS and incubated with RNase in a 37°C water bath for 30 minutes. The cells were subsequently incubated with propidium iodide at 4°C in the dark for 30–60 minutes and analyzed using a flow cytometer (Beckman, USA).

### Apoptosis assay

The apoptosis assays were performed at 48 hours, 72 hours and 96 hours after the cells were infected with the adenovirus. The HepG2 and sk-Hep1 cells infected with Ad-LZAP or Ad-control were washed twice in ice-cold PBS, resuspended in 400 µl of 1× Binding Buffer and incubated with Annexin V-FITC (Bestbio, China) for 15 minutes at 4°C in the dark, according to the manufacturer's instructions. After staining, the cells were incubated with propidium iodide for 5 minutes at 4°C in the dark and then analyzed using a flow cytometer (Beckman, USA).

### Cell migration assay

The cell migration assays were performed in a chamber system consisting of polycarbonate membrane inserts with an 8-µm pore size (Corning, USA) placed in 24-well cell culture insert companion plates. The migration assay was conducted at 48 hours after the HepG2 and sk-Hep1 cells were infected with Ad-control or Ad-LZAP. The cells (5×10^4^ in 100 µl of growth medium without FBS) were placed in the upper chamber and 500 µl of growth medium with 10% FBS was placed in the lower chamber. The cells were incubated at 37°C for 12 hours. Following the incubation, the insert membranes were fixed with 75% methanol for 30 minutes. The cells on the upper surface were removed with cotton-tipped swabs, and the migrated cells on the lower surface were stained with 0.5% crystal violet containing 20% methanol for 60 minutes. The stained cells were counted under an inverted microscope (10 fields per membrane). Each experiment was performed in triplicate.

### Matrigel invasion assay

The Matrigel invasion assay was performed in a chamber system consisting polycarbonate membrane inserts with 8-µm pores (Corning, USA) placed in 24-well cell culture insert companion plates. The inserts were coated with a thin layer of 0.5 mg/ml Matrigel Basement Membrane Matrix (BD Biosciences, Bedford, MA). The invasion assay was conducted at 48 hours after the HepG2 and sk-Hep1 cells were infected with Ad-control or Ad-LZAP. The cells (1×10^5^ in 200 µl of growth medium without FBS) were placed in the upper chamber, and 0.5 ml of growth medium containing 20% FBS was placed in the lower chamber. The cells were incubated at 37°C and allowed to invade through the Matrigel layer for 48 hours. After incubation, the insert membranes were fixed with 75% methanol for 30 hours. The cells on the upper surface were removed with cotton-tipped swabs, and the invading cells on the lower surface were stained with 0.5% crystal violet containing 20% methanol for 60 minutes. The stained cells were counted under an inverted microscope (10 fields per membrane). Each experiment was performed in triplicate.

### Tumorigenicity assays in nude mice

Female BALB/c athymic nude mice (4–5 weeks old) were obtained from the Medical Experimental Animal Center of Guangdong Province. The mice were randomly divided into 4 groups of 8 mice each. Group 1 was injected with HepG2 cells that had been infected with Ad-control; Group 2 was injected with HepG2 cells that had been infected with Ad-LZAP; group 3 was injected with sk-Hep1 cells that had been infected with Ad-control; group 4 was injected with sk-Hep1 cells that had been infected with Ad-LZAP. For the injections, 8×10^6^ tumor cells were suspended in 200 µl PBS and then subcutaneously injected into the posterior flank of the mice. The tumor size was monitored every 3 days by measuring the length (L) and width (W) of the tumor with calipers. The tumor volume was calculated according to the following formula: (L×W^2^)/2. At 4–5 weeks after inoculation, all the mice were sacrificed, and the tumors were harvested and photographed. The weight of the tumors was also measured. All the experimental procedures involving animals were performed in accordance with the Guide for the Care and Use of Laboratory Animals (NIH publications Nos. 80–23, revised 1996) and the institutional ethical guidelines for animal experiments.

### Statistical analysis

The statistical analyses were performed using the Statistical Package for the Social Sciences, version 16.0 (SPSS Inc., Chicago, IL, USA). A paired-samples t-test was used to compare LZAP mRNA and protein expression in the HCC tumors with that of their paired adjacent non-cancerous tissue samples. The correlation between tumor LZAP expression and the clinical and pathological features was performed using χ2 tests. Overall survival curves were calculated with the Kaplan-Meier method and were analyzed with the log-rank test. A Cox proportional-hazards analysis was used in univariate and multivariate analyses to explore the effects of LZAP expression and HCC clinicopathological variables on survival. The results were expressed as mean ± SD and analyzed using the Student's t-test. Differences were considered significant at p<0.05.

## Supporting Information

Figure S1 **LZAP protein expression in normal liver cells (LO2) and the HepG2 and sk-Hep1 cells infected with Ad-LZAP at different titers.** Western blotting showed that the LZAP expression in the HepG2 and sk-Hep1 cells infected with Ad-LZAP at MOIs of 50, 100, 200 and 400 was significantly higher than that of the HepG2 or sk-Hep1 cells infected with the Ad-control. The LZAP expression in the HepG2 and sk-Hep1 cells infected with Ad-LZAP at a MOI of 200 was higher than that in normal liver cells.
(TIF)

Figure S2 **LZAP silencing in the HepG2 and sk-Hep1 cells infected with Ad-LZAP at MOI 200.** Western blotting showed that siLZAP-3# had the highest knockout efficiency of the three siRNAs tested. Therefore, siLZAP-3# was used for all the subsequent experiments.
(TIF)

Figure S3 **LZAP knockout in the HepG2 and sk-Hep1 cells infected with Ad-LZAP at a MOI of 200 and its effect on cell viability.** (**A**) The silencing of LZAP expression in the HepG2 and sk-Hep1 cells infected with Ad-LZAP significantly increased cell proliferation, as assessed by the MTS assay. The proliferation levels were similar in the knockout cells and in the cells infected with the Ad-control. (**B**) The colony formation assays revealed a marked increase in colony number and size after the LZAP knockout in the HepG2 and sk-Hep1 cells infected with Ad-LZAP. Similar values were found for the HepG2 and sk-Hep1 cells infected with the Ad-control. *p<0.05; NS: not significant.
(TIF)
