# Supplementary figures and images for: Identification of LZAP as a New Candidate Tumor Suppressor in Hepatocellular Carcinoma
Source: PLoS One. 2011 Oct 19;6(10):e26608. doi: 10.1371/journal.pone.0026608 (PMC3197520; doi:10.1371/journal.pone.0026608)

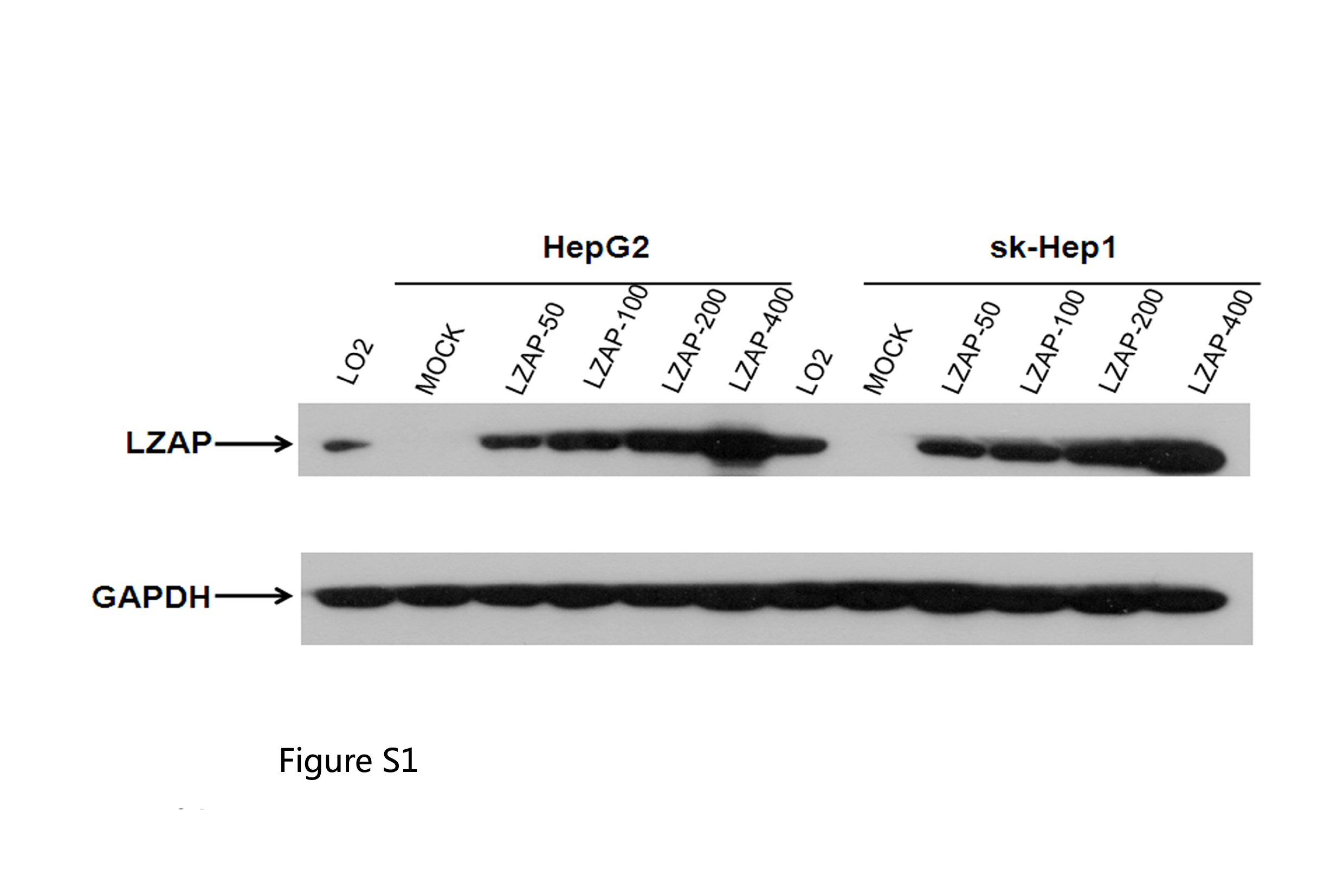

Supplement: Figure S1 — LZAP protein expression in normal liver cells (LO2) and the HepG2 and sk-Hep1 cells infected with Ad-LZAP at different titers. Western blotting showed that the LZAP expression in the HepG2 and sk-Hep1 cells infected with Ad-LZAP at MOIs of 50, 100, 200 and 400 was significantly higher than that of the HepG2 or sk-Hep1 cells infected with the Ad-control. The LZAP expression in the HepG2 and sk-Hep1 cells infected with Ad-LZAP at a MOI of 200 was higher than that in normal liver cells. (TIF) [file pone.0026608.s001.tif]

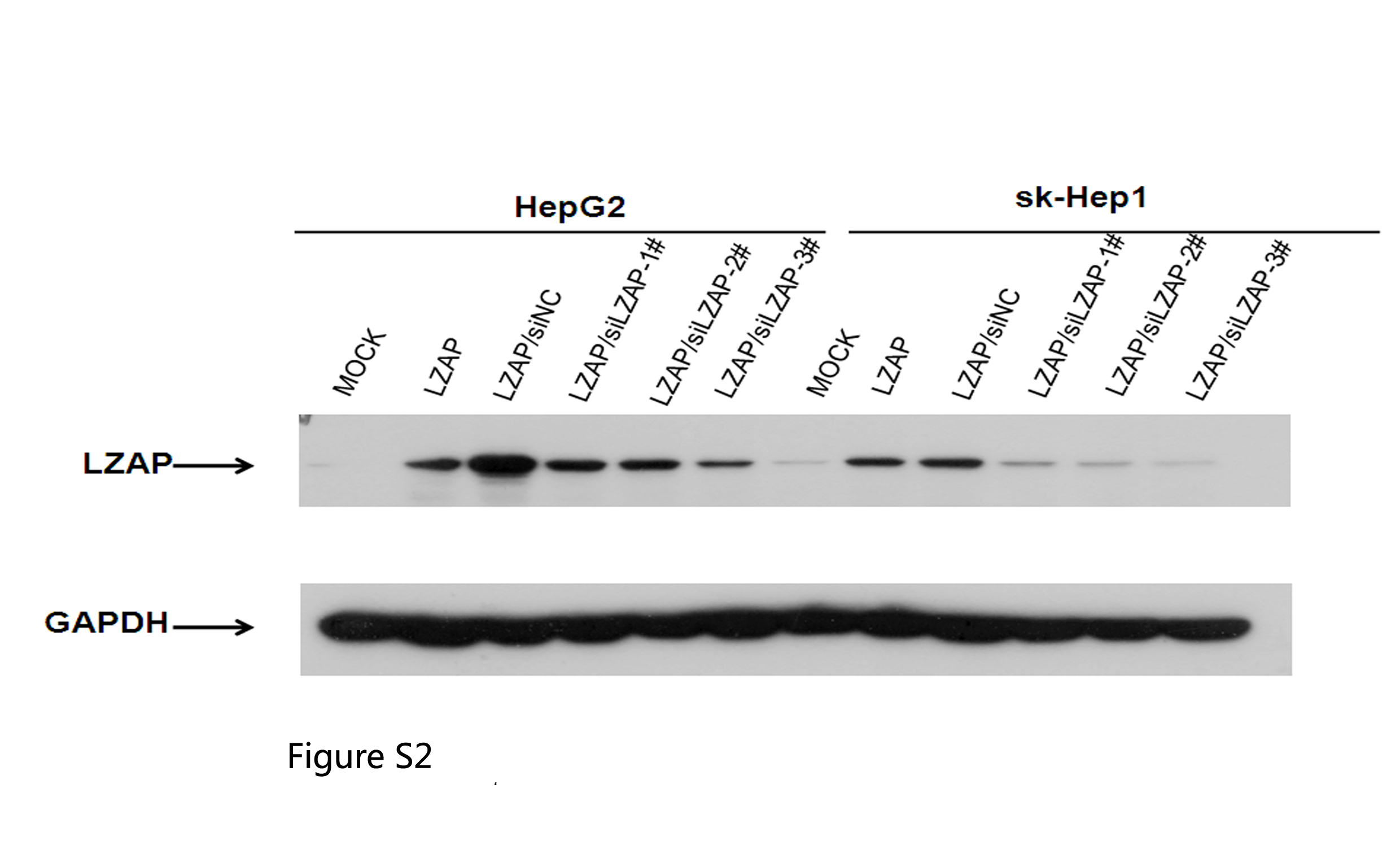

Supplement: Figure S2 — LZAP silencing in the HepG2 and sk-Hep1 cells infected with Ad-LZAP at MOI 200. Western blotting showed that siLZAP-3# had the highest knockout efficiency of the three siRNAs tested. Therefore, siLZAP-3# was used for all the subsequent experiments. (TIF) [file pone.0026608.s002.tif]

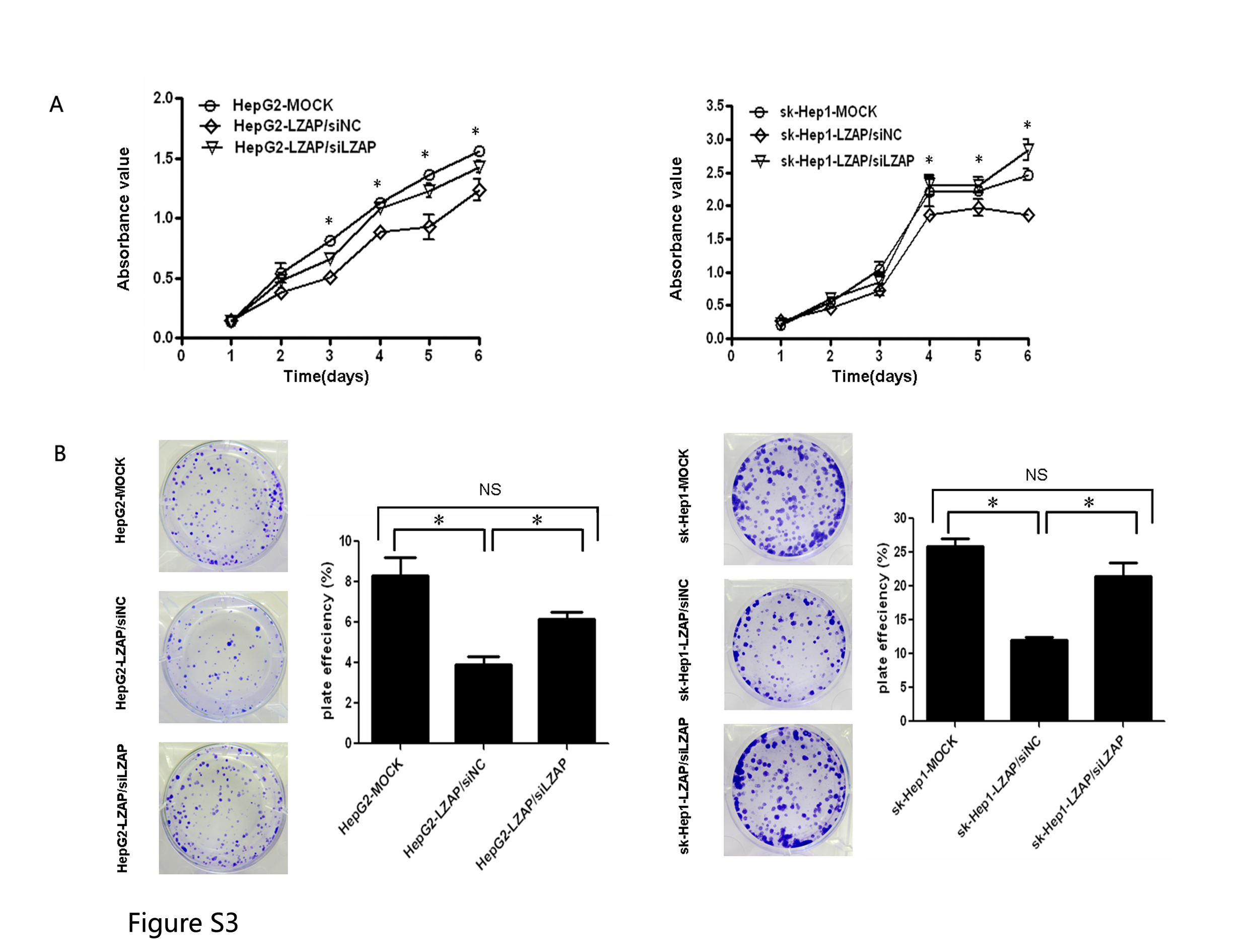

Supplement: Figure S3 — LZAP knockout in the HepG2 and sk-Hep1 cells infected with Ad-LZAP at a MOI of 200 and its effect on cell viability. (A) The silencing of LZAP expression in the HepG2 and sk-Hep1 cells infected with Ad-LZAP significantly increased cell proliferation, as assessed by the MTS assay. The proliferation levels were similar in the knockout cells and in the cells infected with the Ad-control. (B) The colony formation assays revealed a marked increase in colony number and size after the LZAP knockout in the HepG2 and sk-Hep1 cells infected with Ad-LZAP. Similar values were found for the HepG2 and sk-Hep1 cells infected with the Ad-control. *p<0.05; NS: not significant. (TIF) [file pone.0026608.s003.tif]
